# Characterization of collaborative management paths for public health at the county-level government in China: 3 cases based on fsQCA

**DOI:** 10.3389/frhs.2026.1797149

**Published:** 2026-03-19

**Authors:** Jiangping Fu, Rui Hu, Bing Cao, Zhi Sun

**Affiliations:** 1 Nantong Institute of Technology, Nantong, Jiangsu, China; 2 Nanjing Sport Institute, Nanjing, Jiangsu, China; 3 Zhengde Polytechnic, Nanjing, Jiangsu, China; 4 Jiangsu Open University, Nanjing, Jiangsu, China

**Keywords:** characterization, collaborative management paths, county-level government, fuzzy-set qualitative comparative analysis, non-linear structure, policy resources, public health

## Abstract

**Background:**

County-level governments (CLG) are the basic organizational units of China's administrative power. The collaborative management paths (CMP) for public health at the CLG carry a variety of pressures and are responsible for coordinating the allocation of resources, and need to be well developed in terms of their capacity structure.

**Method:**

This study defines the CMP of CLG in public health as six variables of policy resource: configuration capability, perception capability, insight capability, integration capability, learning capability, and innovation capability. This study incorporates the fsQCA algorithm to explore the non-linear relationship between the collaborative management capabilities of the CMP of CLG in public health and policy resources.

**Results:**

A configuration of the CMP of CLG for public health was identified (solution coverage 36.67%, solution consistency 98.24%). The CLG's CMP has full-time-phase characteristics, i.e., the diversion management time-phase is characterized by conventional and non-conventional management time-phase groupings, but the non-conventional management time-phase does not have a bottleneck level. CLG's CMP has 3 core elements (Integration, Learning, and Perception Capabilities) and 2 supporting elements (Innovation and Insight Capabilities). The bottleneck level analysis of CLG's CMP resulted in a 10% level of perceived capacity being required to achieve a 60% level of configured capacity. The sensitivity test of the CMP for CLG suggests that the pathway is robust.

**Conclusion:**

This study presents a framework for observing/interpreting the results of CLG as a managerial behavior (policy resource management) at the grassroots level of government from the perspective of CMP.

## 1 Introduction

The COVID-19 pandemic outbreak has put County-Level Government (CLG) around the world to the test of the Collaborative Management Pathway (CMP) for public health (1). The CLGs are interested in human life and social and economic stability, with the power of system administration to implement CMPs and make their own public health decisions for sustainable human development (2). In Chinese government organizations, CLGs assume responsibility for the administration of grassroots organizations and the collaborative management of public health resources, practicing CMP (3).

In the CMP governance structure, CLGs face limited resources (4), as well as political marginalization (5, 6). In CLG's study of governing competence, scholars of social cognitive theory, proposed a model and path for the synergistic management of self-efficacy (expectations and relationships)-goal-behavior (7, 8). There are shortcomings in the authority of the CLG as the main body of global public health governance as well as in the interactivity of multilevel participation, highlighted by gaps in the CMP (9). The dynamic interaction, interdependence, and mutual influence of cognition and practice in public health (10, 11) together drive and shape CLG's CMP (efficacy-goal-behavior dimension) for resource management. In other words, the management of public health resources by county-level governments is essentially a reflection of their perceptions and behaviors, which are interrelated (12). This requires CLGs to engage in full time-phase planning for CMPs to respond to environmental changes in the course of managing public health crises, routine events (practices) (13). The full TP planning of CLG's CMP exemplifies the effectiveness of the efficacy-goal-behavior dimension for resource management and requires continuous observation and testing.

The issue of “information silos” may be a common phenomenon in CLG's public health CMPs (9, 14, 15). CMP is a hot topic in the field of public health research, favoring the problem of information silos (16). CMPs work together to improve CLG's public management performance with cross-sectoral expertise (17–19). In the context of dynamic systems, the governance of CMPs includes resource, economic, social, cultural, and educational characteristics that shape various conditions and constraints (20–23). Based on different perspectives, various scholars have proposed their own dynamic systems view of CMP: integration-innovation (24), digital transformation (25), and attention allocation (2). The dual perspective of information silos and dynamic systems presents a practical need for sensitivity testing (core elements, bottleneck levels) of CLG's CMP.

On the time-phase (TP) dimension (2), the CMP of public health in CLG can be categorized into, conventional management (CM), non-conventional management (NCM, crisis management, e.g., during the COVID-19 outbreak) and diversion management (DM, coupling of conventional and non-conventional management). CM sub-paths have static characteristics and resource configuration capability (CC) and information synergy mechanism flaws are not prominent (26). The NCM sub-pathway is characterized by dynamics and the relatively prominent dimensions are perception capability (PC), insight capability (InsC), integration capability (IntC), learning capability (LC), and innovation capability (InnC) (1, 3). DM sub-paths are characterized by dynamic and static coupling, and efficiency improvement/capability migration of CM and NCM is achieved through collaborative management of resources (27, 28). The current allocation and emphasis of policy resources by the CLG is heavily dependent on the prioritization and effectiveness of social issues, further characterizing the CMP (29, 30). It has been shown that CLG's CMP for policy resources is a dynamic iterative relationship between interpersonal, motivational and functional elements (31), and claims that each element contributes to public health performance (32). This dynamic iterative relationship creates significant differences in the CLG's CMP and requires continuous observation of its TP characteristics.

Based on the analysis of the above information, the topic of this study is the characterization of the CMP of public health in CLG with an exploratory study and the following hypotheses are made:

H0: Characterization of the socio-cognitive model of CLG's CMP, i.e., what are the configurations of the CMP based on the socio-cognitive model (the way the elements are combined and their robustness).
H1: TP characterization of CLG's CMP for public health, i.e., what are the elements that make up a dynamic system based on the TP dimension.
H2: Sensitivity testing of CLG's CMP for public health, including core elements and bottleneck levels.

## 2 Method

### 2.1 Study design

Our research uses secondary calculations of existing data (cases). However, in order to demonstrate scientific rigor, this study was reviewed by the Medical Ethics Committee of Zhengde Polytechnic (ZD-2025PE-004).

Our research was divided into three steps.

**Figure 1:**
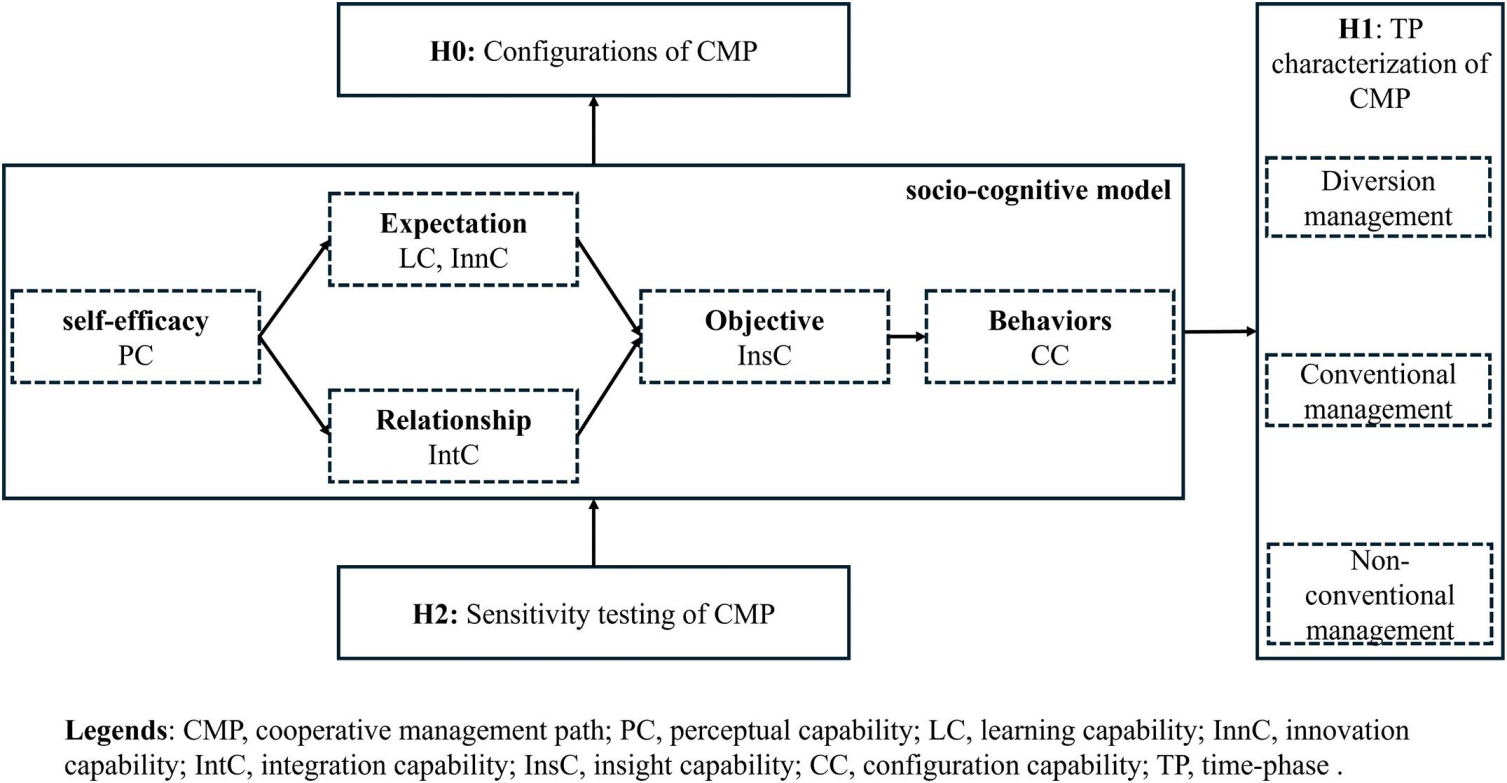
Cooperative management model based on socio-cognitive model.

The first step is to study the precise setting of the hypothesis. See Figure 1. All of our researchers were matched with social cognitive models and public health practices. From the information in the introduction (1–3), it can be seen that the CLG's CMP runs through the entire process of matching social cognitive models and public health practices. At the same time, CLG's CMPs are manifested in three types: routine management, diversion management, and non-routine management.

In the socio-cognitive model, PC is set to represent the self-efficacy element of CLG's CMP, InnC and LC together are set to represent the expectation element of CLG's CMP, IntC is set to represent the relationship element of CLG's CMP, InsC is set to represent the goal element of CLG's CMP, and CC is set to represent the behavioral element of CLG's CMP.

The second step mainly involves researching sample selection and data collection. This study focuses on county-level governments. This study used a case study approach to collect data.

The third step mainly involves researching data calculation and analysis. The main objective of this study was to characterize the CMP of public health in CLG. This study was not intended to be a quantitative/structural equation study between the goals and outcomes of CLG's CMP for public health. This study, based on the information in the preamble section, identified a non-linear logistic relationship between the variables of CLG's public health CMP. This study incorporated fuzzy qualitative comparative analysis (fsQCA 4.1 version) as a method of computing the data to explore the nonlinear relationship between the CMP variables (33). What must be clearly expressed is that this study makes an implicit assumption that the elements and variables of the CMP are the same concept, i.e., the elements are the variables, while at the same time the relationship between the variables is the relationship between the elements.

### 2.2 Research sample and data

This study focuses on CLG. As an administrative organization (institution), CLG in China is the basic administrative units in the entire administrative system. This study incorporates a multi-case study method, using horizontal observation of multiple cases to achieve rigor and scientific accuracy.

Based on the research hypotheses, and taking into account the characteristics of the study population/CLG, this study further refined the indicator system of CMP. CLG's CMP for public health has three TPs for CM, DM and NCM. Also, CLG's CMP for public health has six Level 1 variables of CC, PC, LC, InnC, IntC, and InsC. See Supplementary Table S1.

According to a meta-analysis of the data, CC variables for public health in CLGs were assessed using three indicators: public health unit costs, the number of hospital beds per thousand people, and the number of medical personnel per thousand people (1–4, 34). According to a meta-analysis of the data, PC variables for public health in CLGs include six indicators and nine items related to administrative resources: population, education, culture, sports, economy, and environment (1–4, 34). According to a meta-analysis of the data, LC variables for public health in CLGs include four indicators: crisis communication, emergency training, emergency drills, and professional training (1–4, 34). According to a meta-analysis of the data, InnC variables for public health in CLGs were measured using four indicators: institutional innovation, organizational innovation, technological innovation, and information dissemination innovation (1–4, 34). According to a meta-analysis of the data, IntC variables for public health in CLGs were measured using five indicators: per capita gross domestic product, epidemic prevention and control funding, social assistance, number of employees, and assistance from higher-level governments (1–4, 34). According to a meta-analysis of the data, InsC variables for public health in CLGs were assessed based on four indicators: hazard identification, inventory management, early warning notifications, and measures to be taken (1–4, 34).

A PC variable for Co-Management is set to indicate the TP of the CM of the CLG's CMP for Public Health. The 4 variables LC, InnC, IntC, and InsC of Co-Management are set to indicate the TP of the NCM of the CMP of Public Health of the CLG. A CC for Collaborative Management is set to represent the TP of the DM of the CLG's CMP for Public Health.

Our research data collection is divided into two parts. The first part is derived from previous research, and the second part is derived from the target sample of government websites. See Supplementary Table S1. Wei's team conducted a case study on the dynamic capabilities of three CLGs in China during a public health crisis (3). The four indicators of LC, InnC, IntC, and InsC were incorporated into the evaluation of the CLG of Sixian County, Linshui County, and Jiutai District. The Octopus Collector was used as the data collection tool (35), and detailed evaluation criteria (weights) were established. Finally, the calculated results of the indicator weight percentages were obtained.

The data for the three indicators of the CC of CLG are sourced from the basic information data sections of Sixian County, Linshui County, and Jiutai District. The data for the six indicators of the PC of CLG were obtained from the government websites of Sixian County, Linshui County, and Jiutai District (36–38).

To add and elaborate, the three cases in this study should be representative of the real phenomenon of county-level government in China. The difference between the three cases is the geographical location (Jiutai District is located in the north of China, Si County is located in the center of China, and Linshui County is located in the south of China) (3). Based on the layout of this geographic location, it should satisfy the representativeness of this study. Meanwhile, the county level economic factor (GDP) is an important factor in the CMP of public health, which is 24.35 billion dollars in Jiutai District, 26.74 billion dollars in Linshui County, and 29.324 billion dollars in the Sixian county in 2022 (3).In scientific studies, this small difference (county-level economic factors) is not enough to affect the final results (county-level CMP).

### 2.3 Calculation

Through a meta-analysis of the data in the introduction section, it was found that there is a non-linear relationship between the variables of CLG's CMP. fsQCA has been proven to be an effective method for exploring and verifying nonlinear relationships between variables (33, 39).

This study sets the CC of CLG's CMP as the outcome variable (dependent variable) and the dataset (PC, LC, InnC, IntC, and InsC) as the independent variable. In response to the analysis of the overall causal conditions of socio-cognitive theory (the variables of CLG's CMP), fsQCA can build better causal groupings by combining the core elements through necessary conditions and configurations. This causal configuration is referred to as the CLG's CMP for public health.

The data calculation process consists of seven steps.

The first step is to perform descriptive statistics on the three cases and normalize the collected data (percentage).

The second step is to calibrate the data by calibrating the condition data (independent variable) and result data (dependent variable) using the direct standard method, incorporating calibration standards 0.95 (complete subordination), 0.50 (intersection point), and 0.05 (complete non-subordination) (33, 39).

The third step is to analyze the necessity of individual conditions for the independent variable dataset and include the standard of necessary conditions for consistency (>0.9) (33, 39).

The fourth step is to conduct a sufficiency analysis of the configuration conditions, incorporate the truth table algorithm, and report on the three solutions/configurations (i.e., complex solution, intermediate solution, and simple solution) (33, 39).

The fifth step involves conducting robustness tests, incorporating *post-hoc* robustness testing methods (consistency threshold of 0.75) to avoid randomness and sensitivity in the results (33, 39).

The sixth step involves identifying non-essential conditions, using R language tools, incorporating cross-validation methods, calculating upper limit regression (CR) and upper limit envelope (CE), and incorporating criteria for identifying non-essential conditions [effect size (*d* < 0.1), or significance level (*P* > 0.01), or both] (33, 39).

The seventh step involves conducting a bottleneck level analysis, with the inclusion criteria being the degree of one or more independent variables that require a percentage.

Based on the results of the data calculations, data analysis was performed to characterize the CMP of CLG.

Based on the grouping results of steps 4 and 5, the characteristics of the socio-cognitive model of the CMP of the H0 CLG were determined, incorporating descriptive statistics on the way the elements of the configuration were combined and robustness tests.

Based on the results of steps 4, 5 and 7, the TP characteristics of the CMP of H1CLG were determined. H1 The analysis of the TP characteristics of CLG's CMP firstly incorporated the grouping (high and low levels) of the 5 variables PC, LC, InnC, IntC and InsC as criteria for the qualitative analysis of the TP characteristics, “If all the variables are observed, it is determined that the same managerial pathway has a full TP characteristic, which suggests that the CLG's CMP is comprehensive and indirectly suggests that CLG's CMP is likely to be more effective for resource allocation; otherwise, the reverse is true”, followed by the inclusion of the bottleneck level (percentage) of the 5 variables as a criterion for the quantitative analysis of the TP characteristics (in the form of a 1-variable equation).

Based on the results of Steps 2, 3, 4, and 6, a comprehensive analysis of the elemental characteristics of the sensitivity testing of CMPs for H2 CLGs was conducted, incorporating which elements were core elements as criteria.

Based on the results of step 7, the bottleneck level characterization of the sensitivity test for the CMP of H2 CLG was determined by incorporating the 1-equation as a descriptive statistical criterion.

## 3 Results

### 3.1 Descriptive results

Data for six variables, 26 indicators, and 29 items related to the collaborative management of the CLGs of Sixian County, Linshui County, and Jiutai District were normalised (average percentage values). See Supplementary Table S1.

It should be noted that in order to reduce the numerical differences in the data, the calculation units for the corresponding entries have been adjusted to achieve two decimal places within the range of 0–100 (e.g., percentage values * 100).

**Calibration of Conditions and Results:** Following the steps of fsQCA analysis, this study used the direct calibration method to convert the data into fuzzy set membership scores, with calibration standards of complete membership 0.95, intersection point 0.5, and complete non-membership 0.05 (33, 39).

Supplementary Table S1 shows the calibration information for the various conditions and results in this study.

### 3.2 Characterization of the socio-cognitive model

#### 3.2.1 Configuration

Based on the number of samples (3 CLGs cases) in the existing studies, this study sets the consistency threshold at 0.8, the PRI threshold at 0.75, and the frequency threshold at 1 (33, 39).

Supplementary Table S2 shows the results of the CC analysis (CMP) of CLG.

To be honest, the calculation results of the configuration conditions yielded a total of one intermediate solution (configuration), and this study did not find a simplified solution.

The analysis results show that the consistency level of the CC of CLG (CMP) is 0.98, which is acceptable (>0.7), and the coverage of the plan is 0.37, indicating good data results (33, 39).

CLG's CMP has low PC (∼), high InsC, high IntC, high LC, and high InnC elements and configurations.

#### 3.2.2 Robustness test

Robustness testing was performed by adjusting the consistency threshold of CLG's CMP. The consistency threshold was lowered from 0.80 to 0.75 to avoid randomness and sensitivity in the results of CLG's CMP (33, 39). See Supplementary Table S2.

The adjusted results are consistent with the original results in terms of the state of the set relationship and the fitting parameters. Therefore, the data analysis results (CLG's CMP) of this study are considered to be reliable.

A more intuitive formulation is the robustness of the socio-cognitive model of CLG's CMP.

### 3.3 TP characteristics

Supplementary Table S2 shows the results of the CC configuration (CMP) analysis of the CLG and also indicates the TP characteristics of the CMP of the CLG. From Supplementary Table S3, it can be noticed that the grouping of the CMP of the CLG (INTC*LC*INNC∼PC*INSC), which contains the PC element of the TP of the CM, also contains the four elements of the TP of the NCM (InsC, IntC, LC, and InnC). The configuration of the CLG's CMP illustrates the qualitative characterization of the full TP, i.e., the CLG's CMP is characterized by the full TP (all 5 elements are observed).

### 3.4 Sensitivity testing

#### 3.4.1 Core elements

The core elements of CLG's CMP include three parts: the necessity analysis of individual conditions, the determination of non-necessary conditions, and the sufficiency analysis of the configuration.

##### 3.4.1.1 Necessity analysis of individual conditions

After the anchor calibration of fsQCA is completed, a necessity analysis is performed on individual condition variables.

Supplementary Table S3 shows the results of testing the necessary conditions for the configuration capacity of county-level governments using fsQCA software.

From this, we can see that not all conditions have a consistency level of less than 0.9. Therefore, there are two necessary conditions in the conditional variables (IntC、Lc and InnC). In further analysis, IntC, LC, and InnC must be mentioned first.

##### 3.4.1.2 Determination of non-essential conditions

This study used R version 4.5.1 to test the non-essential conditions for the configuration capabilities of county-level governments.

Supplementary Table S3 shows the non-essential conditions for testing the CC of CLG.

The effect sizes of PC CR0.13 and CE0.25 are both greater than 0.1 (necessary), and the *P*-values CR0.67 and CE0.67 are both greater than 0.01 (not necessary) (33, 39).

This suggests that PC may be either necessary or non-essential for the CMP of CLG.

The other four variables (IntC, LC, InnC, and InsC) are all non-essential for the CMP of the CLG.

##### 3.4.1.3 Sufficiency analysis of the configuration

Based on the results of the intermediate solution of the grouping, the necessity test, and the non-necessity condition test, this study conducted a sufficiency analysis of the configuration of the CMP for CLG.

This study identified 3 core elements (IntC, LC, and PC) and 2 supporting conditions (InnC and InsC) for CMP in CLG.

#### 3.4.2 Bottleneck level testing

Supplementary Table S3 shows the bottleneck level test of the CC (CMP) of CLG.

CMP of CLG need to achieve a 60% CC level for public health/coordinated management, requiring a 10% PC level, while InsC, IntC, LC, and InnC do not have bottleneck levels.

CLG's CMP requires a 50% level of PC to achieve a 100% level of CC, while InsC, IntC, LC, and InnC still do not have a bottleneck level.

Bottleneck level equation for CLG's CMP: 100% CC = 50% PC + 50%?.

Supplementary Table S3 represents the results of the bottleneck level test for the CLG's CC and also the TP characteristics of the CLG's CMP. From the table, it can be observed that 100% level of TP for DM requires 50% TP for CM. Also, 100% level DMTP does not require any TP of NCM. This phenomenon illustrates that the full TP characteristics of the existing CLG's CMP do not have the bottleneck level of the NCM's TP. Expressed as a formula, 100% TP for DM (CC) = 50% TP of CM (PC) + 50% TP of NCM (excluding InsC, IntC, LC, and InnC).

## 4 Discussion

### 4.1 Characteristics of CMP

This study identified one CMP for public health in CLG in China (INTC*LC*INNC∼PC*INSC). Components of the collaborative management path between low PC and CMP of CLG were observed. This phenomenon expresses the possibility that low PC as an independent variable is associated with a strain (CC) of CMP in CLG. The PC encompasses the phenomenon of CLG's synergistic management of resources such as population, economy, education, culture, sports and environment. The low PC of CLG may indicate low attention in the process of collaborative management of resources (2). The high PC (high attention) of CLG's management of public health resources was not detected. In the socio-cognitive model, the low PC illustrates that the CMP of CLG has low self-efficacy. This may validate the political marginalization and information silos of CLG's collaborative management of public health.

An interesting phenomenon is that when validating NCA, 60% of the CC requires 10% of the PC to support it. This may indicate that PC is a prerequisite for the CCy of CLG to coordinate public health management. This may also indicate that PC have been noticed in the current collaborative management process of CLG, but they are receiving low attention (2). At the same time, low attention to PC may be a key factor in the coordinated management of public health by CLG. This may directly validate the efficacy-goal-behavior dimension of the socio-cognitive model, and further may illustrate the characterization of the PC as the logical starting point element of the CMP of the CLG.

The full TP characterization at the CMP demonstrated that high LC, high InnC, high IntC, and high InsC were observed simultaneously. This suggests that the CC of the CMP for public health in CLG may be associated with both LC, InnC, IntC and InsC. These four capabilities, in turn, have been defined by scholars as the CLG's dynamic capacity for crisis public health and the TP of the CLG's public health/co-managed NCM (3, 34). This may be direct evidence that CLG's CMP is relatively comprehensive. This may indirectly prove that CLG's CMP may be effective for CC of resources. However, the results of the bottleneck level test also observed the phenomenon of no bottleneck level in the TP of the NCM of the CLG's CMP, i.e., the bottleneck level of the four elements of the TP of the NCM (LC, InnC, IntC, and InsC) is zero. This suggests that the TP (CC) of the CLG's public health/co-management DM may be related to both the TP of the CM and the TP of the NCM. This seems to be indirect evidence of the attention deficit phenomenon that scholars agree CLGs have at CMPs in public health (1–3).

### 4.2 Advantages and limitations

**Advantages:** Collaborative management of public health by CLG is a hot topic. The Chinese government has successfully achieved SDG 4 (zero hunger) within the framework of sustainable development through its systematic administrative power, demonstrating its unique capabilities. This study uses a multi-case empirical approach to validate the collaborative management of public health (social cognitive model) by county-level governments in China. The fsQCA algorithm is an advantage for the computation of multi-factors for multiple cases. To express more clearly, a fuzzy dataset is validated by fsQCA for the phenomenon of success or failure of multiple cases with multiple factors in a transversal direction. And this success or failure case may have a universal value and may have the value of being cited internationally.

**Limitations:** This study is based on secondary data calculations. Although we supplemented the data and normalized it, this can only represent the results of this data calculation. FsQCA is applicable to several case studies, but the sample size of the three cases in this study is limited. More cases or data calculations of qualitative or mixed-methods may differ from the results of this study.

## 5 Conclusion

This study presents a framework for observing/interpreting the results of CLG as a managerial behavior (policy resource management) at the grassroots level of government from the perspective of CMP. The currently observed characteristics of CMPs expose the strengths and limitations of existing CLGs for policy resource management behavior.

## Data Availability

The original contributions presented in the study are included in the article/Supplementary Material, further inquiries can be directed to the corresponding author.
