# Supplementary material for: Characterization of collaborative management paths for public health at the county-level government in China: 3 cases based on fsQCA
Source: Front Health Serv. 2026 Mar 19;6:1797149. doi: 10.3389/frhs.2026.1797149 (PMC13044139; doi:10.3389/frhs.2026.1797149)
Supplement: Supplementary file 1 [file table1.docx]

Supplementary Material

# Supplementary Table 1

**TABLE 1. Variables in the collaborative management model.**

**TABLE 1(A). Statistical table of outcome variables (configuration capabilities).**

| **Sample** | **Indicators** |  |  | **Configuration capabilities** |
| --- | --- | --- | --- | --- |
| **Case** | **Unit cost** | **Number of beds** | **Number of medical staff** |  |
| Sixian County | 15.13 | 59 | 6 | 26.71 |
| Linshui County | 15.82 | 49 | 49 | 37.94 |
| Jiutai District | 14.18 | 55 | 55 | 41.39 |

**Remarks:** Unit cost: Public health unit cost, statistical unit (yuan); Number of beds: Number of hospital beds per thousand people, statistical unit (percentage * 10,000); Number of medical staff: Number of medical staff per thousand people, statistical unit (percentage * 1,000).

**TABLE 1(B). Statistical table of conditional variables (configuration capabilities).**

| **Sample** | **Indicators** | | | | | | | | | **CC** |
| --- | --- | --- | --- | --- | --- | --- | --- | --- | --- | --- |
| **Case** | TP | PAS | PD | Edu | CT | Spo | E1 | E2 | Env |  |
| SC | 76.33 | 58.10 | 41.10 | 24.20 | 63.20 | 7.60 | 89.28 | 293.24 | 80.00 | 81.45 |
| LC | 70.75 | 57.94 | 37.10 | 19.39 | 33.60 | 23.46 | 78.82 | 267.4 | 92.88 | 75.71 |
| JD | 56.99 | 64.60 | 16.90 | 11.34 | 53.70 | 63.51 | 105.88 | 243.5 | 86.03 | 78.05 |

**Remarks:** SC, Sixian County; LC, Linshui County; JD, Jiutai District; CC, Configuration capabilities; TP, Total population, in ten thousand; PAS, population age structure, proportion of population aged 15–59 and above; PD, population density, number of people per square kilometers/10; Edu, Education, primary education population structure, proportion of population aged 14 and under; CT, Culture and Technology, number of patent grants, 2023 quantity/10; Spo, Sports, annual number of people participating in sports, 2023 participation rate percentage; E1,Economy (administrative cost per unit), RMB 100/person; E2,Economy (GDP), GDP (Gross Domestic Product), RMB 100 million; Env, Environment, number of days with good air quality in 2023/365*100.

**TABLE 1(C). Statistical table of conditional variables (insight capability).**

| **Sample** | **Indicators** |  |  |  | **IC** |
| --- | --- | --- | --- | --- | --- |
| **Case** | **IHS** | **MI** | **EWN** | **MT** |  |
| Sixian County | 25 | 0 | 25 | 25 | 22.5 |
| Linshui County | 25 | 25 | 15 | 15 | 24 |
| Jiutai District | 25 | 0 | 15 | 0 | 12 |

**Remarks:** IC, Insight capability; IHS, Identification of Hazard Sources; MI, Material inventory; EWN, Early warning notification; MT, Measures to be taken; Statistical unit, percentage.

**TABLE 1(D). Statistical table of conditional variables (integration capability).**

| **Sample** | **Indicators** |  |  |  |  | **IC** |
| --- | --- | --- | --- | --- | --- | --- |
| **Case** | PCGDP | EPCF | SA | NS | SGA |  |
| Sixian County | 20 | 10 | 20 | 10 | 20 | 24 |
| Linshui County | 10 | 20 | 10 | 0 | 20 | 18 |
| Jiutai District | 0 | 0 | 0 | 20 | 20 | 12 |

**Remarks:** IC, integration capability; PCGDP, per capita gross domestic product; EPCF, Epidemic prevention and control funds; SA, Social assistance; NS, Number of staff; SGA, Superior government assistance; Statistical unit, percentage.

**TABLE 1(E). Statistical table of conditional variables (learning capability).**

| **Sample** | **Indicators** |  |  |  | **LC** |
| --- | --- | --- | --- | --- | --- |
| **Case** | CC | ET | ED | PT |  |
| Sixian County | 25 | 15 | 25 | 25 | 18 |
| Linshui County | 25 | 0 | 25 | 25 | 15 |
| Jiutai District | 25 | 0 | 15 | 0 | 8 |

**Remarks:** LC, learning capability; CC, Crisis Communication; ET, Emergency Training; ED, Emergency drills; PT, Professional Training; Statistical unit, percentage.

**TABLE 1(F). Statistical table of conditional variables (innovation capability).**

| **Sample** | **Indicators** |  |  |  | **IC** |
| --- | --- | --- | --- | --- | --- |
| **Case** | II | OI | TI | IID |  |
| Sixian County | 25 | 25 | 25 | 25 | 20 |
| Linshui County | 25 | 25 | 25 | 25 | 20 |
| Jiutai District | 0 | 25 | 25 | 25 | 15 |

**Remarks:** IC, innovation capability; II, Institutional innovation; OI, Organizational Innovation; TI, Technological innovation; IID, Innovation in Information Dissemination; Statistical unit, percentage.

**TABLE 1 (G). Calibration anchors for condition variables and result variables**.

| **Variables** |  | **Target set** | **Completely subordinate** | **Intersection point** | **Completely independent** |
| --- | --- | --- | --- | --- | --- |
| **Classification** | **Name** |  | **Affiliation degree 0.95** | **Affiliation degree 0.50** | **Affiliation degree 0.05** |
| Outcome variables |  |  |  |  |  |
|  | configuration capability | High configuration capability | 41.05 | 37.95 | 27.83 |
| Conditional variables |  |  |  |  |  |
|  | perception capability | High perception capability | 81.11 | 78.06 | 75.94 |
|  | insight capability | High insight capability | 23.85 | 22.50 | 13.05 |
|  | integration capability | High integration capability | 23.40 | 17.89 | 12.60 |
|  | learning capability | High learning capability | 17.70 | 14.87 | 8.70 |
|  | innovation capability | High innovation capability | 20.00 | 20.00 | 15.50 |
