# Supplementary material for: Characterization of collaborative management paths for public health at the county-level government in China: 3 cases based on fsQCA
Source: Front Health Serv. 2026 Mar 19;6:1797149. doi: 10.3389/frhs.2026.1797149 (PMC13044139; doi:10.3389/frhs.2026.1797149)
Supplement: Supplementary file 2 [file table2.docx]

Supplementary Material

# Supplementary Table 2

**TABLE 2. Characteristics of collaborative management paths in county-level government.**

| **Conditional variables** | **Condition configuration** | **Time-phase** |
| --- | --- | --- |
| perception capability | ⊕ | conventional management |
| insight capability | ● | non-conventional management |
| integration capability | ● |  |
| learning capability | ● |  |
| innovation capability | ● |  |
| raw coverage | 0.366667 | |
| unique coverage | 0.366667 | |
| consistency | 0.982143 | |
| solution coverage | 0.366667 | |
| solution consistency | 0.982143 | |

**Remarks:** ● = core condition present; ⊕ = core condition absent.
