# Supplementary material for: Characterization of collaborative management paths for public health at the county-level government in China: 3 cases based on fsQCA
Source: Front Health Serv. 2026 Mar 19;6:1797149. doi: 10.3389/frhs.2026.1797149 (PMC13044139; doi:10.3389/frhs.2026.1797149)
Supplement: Supplementary file 3 [file table3.docx]

Supplementary Material

# Supplementary Table 3

**TABLE 3. Sensitivity testing of collaborative management paths in county-level government.**

**TABLE 3 (A). Analysis of Necessity of Configuration Capabilities for County-level Governments**.

| **Conditional variables** | **High configuration capability** | | **Low configuration capability** | |
| --- | --- | --- | --- | --- |
|  | **Consistency** | **Coverage** | **Consistency** | **Coverage** |
| High perception capability | 0.373333 | 0.373333 | 0.686667 | 0.686667 |
| Low perception capability | 0.686667 | 0.686667 | 0.373333 | 0.373333 |
| High insight capability | 0.373333 | 0.373333 | 0.686667 | 0.686667 |
| Low insight capability | 0.686667 | 0.686667 | 0.373333 | 0.373333 |
| High integration capability | 0.373333 | 0.370861 | 1.000000* | 0.993378 |
| Low integration capability | 0.993333* | 0.373333 | 0.366667 | 0.369127 |
| High learning capability | 0.980000* | 1.000000 | 1.000000* | 0.980392 |
| Low learning capability | 0.373333 | 0.373333 | 0.353333 | 0.360544 |
| High innovation capability | 0.366667 | 0.283505 | 0.986667* | 0.762887 |
| Low innovation capability | 0.693333 | 0.981132 | 0.073333 | 0.103774 |

**Remarks:** *, necessary condition, consistency ≥ 0.9.

**TABLE 3 (B). Testing non-essential conditions for configuration capabilities**.

| **Conditions** | **Method** | **c-accuracy** | **Ceiling zone** | **Scope** | **Effect size** | **p-value** | **verification** |
| --- | --- | --- | --- | --- | --- | --- | --- |
| PC | CR | 100% | 0.1100 | 1.000 | 0.1250 | 0.6670 | Possibly necessary |
|  | CE | 100% | 0.2210 | 1.000 | 0.2500 | 0.6670 |  |
| INSC | CR | 100% | 0.0000 | 0.88 | 0.0000 | 1.0000 | Not necessary |
|  | CE | 100% | 0.0000 | 0.88 | 0.0000 | 1.0000 |  |
| INTC | CR | 100% | 0.0000 | 0.88 | 0.0000 | 1.0000 | Not necessary |
|  | CE | 100% | 0.0000 | 0.88 | 0.0000 | 1.0000 |  |
| LC | CR | 100% | 0.0000 | 0.88 | 0.0000 | 1.0000 | Not necessary |
|  | CE | 100% | 0.0000 | 0.88 | 0.0000 | 1.0000 |  |
| INNC | CR | 100% | 0.0000 | 0.88 | 0.0000 | 1.0000 | Not necessary |
|  | CE | 100% | 0.0000 | 0.88 | 0.0000 | 1.0000 |  |

**Remarks:** PC, perception capability; INSC, insight capability; INTC, integration capability; LC, learning capability; INNC, innovation capability; Not necessary, d<0.1and P>0.01; Not necessary，d≧0.1and P≤0.01; Possibly necessary，d≧0.1or P≤0.01.

**TABLE 3 (C). Bottleneck level test for configuration capabilities**.

| **configuration capability** | **perception capability** | **insight capability** | **integration capability** | **learning capability** | **innovation capability** |
| --- | --- | --- | --- | --- | --- |
| **full time-phase** | **conventional management** | **non-conventional management** | | | |
| 0 | NN | NN | NN | NN | NN |
| 10 | NN | NN | NN | NN | NN |
| 20 | NN | NN | NN | NN | NN |
| 30 | NN | NN | NN | NN | NN |
| 40 | NN | NN | NN | NN | NN |
| 50 | NN | NN | NN | NN | NN |
| 60 | 10.0 | NN | NN | NN | NN |
| 70 | 20.0 | NN | NN | NN | NN |
| 80 | 30.0 | NN | NN | NN | NN |
| 90 | 40.0 | NN | NN | NN | NN |
| 100 | 50.0 | NN | NN | NN | NN |
